# Accelerating evidence generation to implementation: Establishment of the Leading Awareness to Action through Implementation of Cardiometabolic Efforts (LATTICE) consortium

**DOI:** 10.1016/j.ajpc.2025.101358

**Published:** 2025-11-22

**Authors:** Laney K. Jones, Ty J. Gluckman, Ankeet S. Bhatt, Leandro Boer, Marc P. Bonaca, Gemme Campbell-Salome, Erica Davis, Nihar R. Desai, Jyothis George, Lisa Head, Francoise A. Marvel, Marc Penn, Eric D. Peterson, Benjamin M. Scirica, Nishant P. Shah, Katherine Wilemon, Bethany A. Kalich, Seth S. Martin

**Affiliations:** a Amgen Inc., One Amgen Center Drive, Thousand Oaks, CA, USA; b Providence Heart Institute, Providence Health System, Portland, OR, USA; c Kaiser Permanente Northern California, Oakland, CA, USA; d Stanford School of Medicine, Palo Alto, CA, USA; e CPC Clinical Research, University of Colorado School of Medicine, Denver, CO, USA; f Yale School of Medicine, New Haven, CT, USA; g Johns Hopkins University School of Medicine, Baltimore, MD, USA; h Summa Cardiovascular Institute, Akron, OH, USA; i UT Southwestern Medicine Center, Department of Medicine, Division of Cardiology, TX, USA; j Brigham and Women's Hospital and Harvard School of Medicine, Boston, MA, USA; k Duke University School of Medicine, Durham, NC, USA; l Family Heart Foundation, Fernandina Beach, FL, USA

**Keywords:** Cardiometabolic, Implementation science, Consortium

## Abstract

Recent scientific advancements have led to the availability of innovative therapies to reduce the risk of cardiometabolic events, offering new tools to address the world’s leading cause of mortality. The length of time from evidence generation to implementation in healthcare settings remains far from optimal, resulting in missed opportunities to improve morbidity and mortality and unrealized population-level benefit. Established in 2023, the Leading Awareness to Action through Implementation of Cardiometabolic Efforts (LATTICE™) consortium’s mission is to unite partners dedicated to improving cardiometabolic health by creating an inclusive platform for sharing evidence-based tools, methodologies, and strategies to address gaps in care at scale. The LATTICE consortium aims to accomplish this by 1) exploring novel strategies for translating evidence-based advancements across diverse clinical practice settings and 2) disseminating successful strategies through a unique collaboration among clinicians, healthcare systems, payors, patients, patient advocacy groups, and life science companies. The LATTICE consortium is prioritizing projects that rigorously test strategies grounded in implementation science and hold significant promise for uptake at scale. Success of the LATTICE consortium will be assessed through improved awareness, access, and implementation of effective, scalable, and sustainable health care strategies that address cardiometabolic gaps in care.

## 1 Introduction

Cardiovascular disease has remained the leading cause of death in the United States for more than a century, with one person dying every 34 seconds [1,2]. Scientific advancements have led to innovative therapies that reduce the risk of cardiometabolic events (e.g., heart attack, stroke), offering new tools to address the world’s leading cause of mortality. Despite this, the full benefit of such therapies have not been readily translated into clinical practice, creating a gap between knowledge and action, with missed opportunities to further reduce risks related to cardiometabolic disease [3]. Not only do these conditions, which include obesity, dyslipidemia, hypertension, diabetes mellitus, chronic kidney disease, metabolic dysfunction-associated steatotic liver disease, and metabolic syndrome [4], significantly impact patients, clinicians, and health systems, they are also influenced by health disparities and meaningfully contribute to rising health care costs [2,5].

Healthcare organizations, clinicians, payers, and life sciences organizations have largely approached cardiometabolic disease in siloes without consistent cross-collaboration and when present, less than optimal partnerships [6]. This fragmented and disjointed approach has likely limited implementation of evidence-based care, exacerbated care gaps, driven inequities in care delivery, and hindered dissemination of learnings that could be scaled in other settings [7]. Rigorously testing multi-level approaches to accelerate the translation of evidence-based advancements into clinical practice has the potential to close gaps in cardiometabolic care and improve the outcomes of patients with these conditions [8,9]. As a discipline, implementation science has the ability to establish and accelerate scalable and sustainable interventions aimed at solving key problems in cardiometabolic care [10,11]. Combining implementation science with improved dissemination efforts could accelerate clinical implementation of evidence-based practices. Accordingly, this report describes the **L**eading **A**wareness **T**o action **T**hrough **I**mplementation of **C**ardiometabolic **E**fforts (LATTICE™) consortium’s inception and ways in which it aims to refine how healthcare addresses cardiometabolic care gaps.

## 2 The LATTICE™ consortium

To address ongoing and prevalent cardiometabolic care opportunities across healthcare systems, the LATTICE™ consortium established a forum by which experts in cardiometabolic medicine and representatives from the life sciences company, (Amgen was the first partner but the consortium envisions multiple partners in the future), could come together to improve upon ways in which cardiometabolic care is delivered. The group had a shared vision of improving the outcomes for those living with cardiometabolic disease and overcoming current obstacles to effective care by:

1) accelerating awareness and adoption of processes to address larger population health burdens,
2) improving the speed by which clinical evidence is incorporated into clinical practice, and
3) reducing siloed care that promulgates ineffective communication between key stakeholders.

**Fig. 1:**
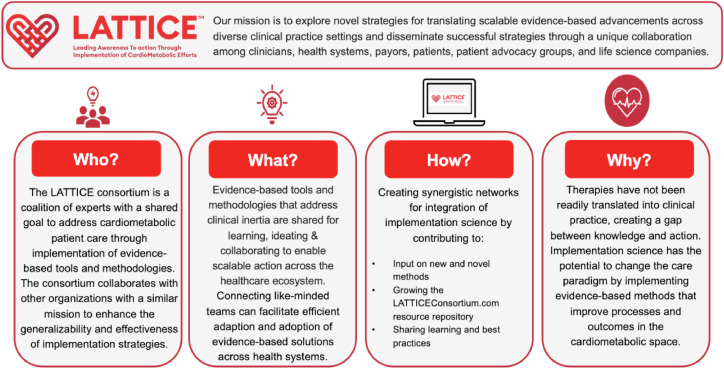
LATTICE™ consortium vision and function.

Thus, the LATTICE™ consortium was created to unite partners seeking to improve cardiometabolic health through establishment of an inclusive platform for sharing evidence-based tools, methodologies, and strategies to address gaps in care at scale. The LATTICE™ consortium aims to accomplish this by 1) exploring novel strategies for translating scalable evidence-based advancements across diverse practice settings and 2) disseminating successful strategies through a unique collaboration between clinicians, healthcare systems, payors, patients, patient advocacy groups, and life science companies. The vision and function of the LATTICE™ consortium are depicted in the Fig. 1.

The LATTICE™ consortium strives to connect with key stakeholders involved in healthcare delivery to reimagine and redefine what partnerships in cardiometabolic health could look like. While other consortiums have served as an inspiration (e.g., Asian Pacific Cardio Metabolic Consortium, https://apcmc.net/ and the Cardiovascular Quality Improvement,Care Innovation Consortium [12]), it was felt that the persistent gaps in cardiometabolic care warrant additional efforts and the LATTICE consortium could be complementary voluntary and inclusive community. LATTICE stands out as a result of its explicit focus on implementation science as a primary scientific framework for advancing cardiometabolic care. The academic-industry partnership also positions LATTICE to potentially achieve what neither sector has been able to do efficiently alone. While Amgen was the first life sciences partner, there is a desire for other industry partners to join the consortium over time.

There is also an intent to invite additional experts with complementary skillsets to join the consortium over time. Experts that participate in the LATTICE™ consortium do so on a voluntary basis; they are not compensated for their involvement. Meetings are held quarterly in person or virtually to provide input and guidance on the development of programs and activities that satisfy current goals. Decisions are made by consensus, with input provided during or in between meetings.

The consortium’s website, www.latticeconsortium.com, serves as the central communication tool to provide open access to ongoing initiatives, key learnings, network of LATTICE™ consortium experts, and scalable solutions. In order for a project, tactic, or resource to be added to the website, however, it must be endorsed by a majority of experts. The website also serves as a means for individuals to submit proposals with strategic alignment, for experts to provide feedback, and for funding partners to perform a review.

## 3 Initial learnings

To date, seven projects have been highlighted by the LATTICE™ consortium that leverage key implementation science principles, aimed at enhancing generalizability and dissemination (more details are available on www.latticeconsortium.com). Thus far, these have been focused on guideline implementation related to hypercholesterolemia.

Each of these projects involve multiple sites, test novel implementation strategies (selected from a pre-defined compilation of validated strategies) [13], specifically define these strategies in order to ensure future replication [14], and measure implementation outcomes through use of an evaluation framework. There are nine categories of strategies: 1) change in care team model/treatment protocol, 2) quality initiative/financial incentives, 3) monitor and address care gaps, 4) information technology electronic health record strategies, 5) tracking and predicting gaps in care, 6) stakeholder education, 7) care coordination, 8) scaling solutions, and 9) engage patients. On average, these projects have addressed 6 multi-level barriers and tested 3 strategies to improve care. While the projects have varied in terms of the patient populations studied, the clinical stakeholders involved, and the duration of follow-up, endpoints of interest have been similar, including rates of low-density lipoprotein cholesterol (LDL-C) testing, LDL-C goal attainment, and uptake of guideline-based care components. Importantly, implementation outcomes are being measured similarly across the projects using implementation theories, models, and frameworks. The consortium plans to apply the well-established RE-AIM framework [15] to examine the impact of implementation of evidence-based practices on patients, clinicians, and health systems (Table). It is anticipated that use of such an approach will allow for more robust comparison of learnings across projects.

**Table 1:** RE-AIM framework.

Reach	Defined as the number and representativeness of the patients affected
Effectiveness	Defined as the impact on health outcomes that are important
Adoption	Defined as the clinician uptake of the implementation strategy
Implementation	Defined as how the implementation strategy is adapted for other health contexts and the implementation costs associated with building and enacting the implementation strategies
Maintenance	Defined as the sustainability of the evidence-based intervention and supportive processes within the health system that allow the program to persist once the initial initiative has ceased

Using the RE-AIM framework to help guide outcome measurement and tracking, we have reached 45 Integrated Delivery Networks and 8,100 clinics including those who have participated in implementation science studies. Among completed projects, improvements have been observed in LDL-C testing, prescription of lipid-lowering therapy, and LDL-Cgoal attainment. Over 35,400 clinicians have participated in projects or studies. Going forward, we intend to examine implementation adoption, cost, and maintenance, as well as impacts on health care.

While incorporation of implementation science into these care improvement efforts is paramount, there is also an important need to reach and engage individuals interested in adopting key learnings. Three key communication channels that can be used for dissemination include:

• The LATTICE™ consortium website,
• The network of LATTICE™ consortium experts, and
• The field teams of life sciences companies

Materials posted on the LATTICE™ website are intended to educate clinicians about implementation tools designed and tested to address specific healthcare gaps. For example, among the various resources on the website are prompts/reminders for patient identification, educational material for clinicians and patients, information about care team models (e.g., lipid navigator), and performance dashboards.

LATTICE™ consortium experts also play an important role in helping to connect learning healthcare systems that may have a shared interest in evaluating strategies to enhance scalability and generalizability. Importantly, these experts can support distribution of information and interventions by publishing, presenting, and/or sharing learnings through educational forums (e.g., scientific congresses). Finally, participating life sciences companies can leverage their field-facing teams to educate healthcare systems about implementation science initiatives.

## 4 Discussion and next steps

As the LATTICE™ consortium works to improve upon the care of patients with cardiometabolic disease, it must adapt to a continuously evolving healthcare environment. Its operating principles that were implemented in 2024 will likely need to change alongside shifts in healthcare delivery. Furthermore, there is a need to accommodate variability in how healthcare is currently provided across healthcare systems, with anticipated differences in willingness to adopt digital health solutions, novel implementation methods, and partnerships with outside organizations. This will likely require expanded expertise in the consortium.

The consortium will continue to prioritize a diversified expertise in caring for various cardiometabolic conditions and populations. Currently, the LATTICE™ consortium is working closely with other organizations focused on prioritizing health equity in cardiometabolic health, including the Association of Black Cardiologists (ABC). Collaborating with other organizations with a similar mission will enhance the generalizability and effectiveness of implementation strategies. Projects prioritized by the consortium will also report on social determinants of health in order to more fully address diverse populations and help improve our understanding of which strategies may help better enhance health outcomes for certain patient groups. This work will also help to minimize efforts that unknowingly increase health disparities.

Although initial efforts have been focused on LDL-C, expansion to other areas with unmet cardiometabolic health needs (e.g., obesity, diabetes mellitus, hypertension, elevated lipoprotein(a), hypertriglyceridemia) is planned. Work related to dissemination will also need to evolve, as educational tools and resources to improve cardiometabolic care are developed. Despite this, there is an intent to maintain the RE-AIM framework, in order to ensure that findings are generalizable and can be replicated across healthcare systems and settings. Importantly, there exists a plan to share all learnings from these initiatives – both successes and failures – along with associated implementation costs. These findings will help others determine the relative value of such efforts. To have a scalable impact, playbooks for implementation (including tools and resources) must also be provided.

We recognize that this work comes with numerous challenges. First, evaluation of some implementation outcomes (maintenance, scalability, and generalizability) may require long-term study. Second, sustainability and dissemination efforts are likely to be limited without ongoing engagement by experts, industry collaborators, and healthcare systems. Third, it will be important to track the use of implementation tools and their relative impact. Fourth, this work is likely to be more consequential as it expands to include an array of cardiometabolic conditions. Finally, while current efforts focus on interventions and strategies carried out at the clinician and healthcare system level, it will be extremely important to have complementary patient-facing tools and resources to address knowledge, attitudes, and behaviors in those we care for.

## 5 Conclusions

The LATTICE™ consortium is a relatively new initiative seeking to unite partners around improved cardiometabolic care. Its success hinges on accelerated dissemination of evidence-based practices across different clinical care settings leveraging implementation science methodology and tools. Such efforts require solutions that are scalable and sustainable in order to close care gaps and improve cardiometabolic health.

## Funding

Amgen has provided administrative support for the LATTICE consortium since launch. Amgen independently has and may continue to provide funding for certain research projects endorsed by LATTICE.

## Declaration of generative AI in scientific writing

Not applicable to this manuscript.

## Data statement

All data is available in this manuscript.

## CRediT authorship contribution statement

**Laney K. Jones:** Writing – review & editing, Writing – original draft, Visualization, Project administration, Methodology. **Ty J. Gluckman:** Writing – review & editing, Writing – original draft, Visualization, Methodology, Conceptualization. **Ankeet S. Bhatt:** Writing – review & editing, Methodology, Conceptualization. **Leandro Boer:** Writing – review & editing, Methodology, Conceptualization. **Marc P. Bonaca:** Writing – review & editing, Methodology, Conceptualization. **Gemme Campbell-Salome:** Writing – review & editing, Writing – original draft, Visualization, Methodology, Conceptualization. **Erica Davis:** Writing – review & editing, Methodology, Conceptualization. **Nihar R. Desai:** Writing – review & editing, Methodology, Conceptualization. **Jyothis George:** Writing – review & editing, Methodology, Conceptualization. **Lisa Head:** Writing – review & editing, Methodology, Conceptualization. **Francoise A. Marvel:** Writing – review & editing, Methodology, Conceptualization. **Marc Penn:** Writing – review & editing, Methodology, Conceptualization. **Eric D. Peterson:** Writing – review & editing, Methodology, Conceptualization. **Benjamin M. Scirica:** Writing – review & editing, Methodology, Conceptualization. **Nishant P. Shah:** Writing – review & editing, Methodology, Conceptualization. **Katherine Wilemon:** Writing – review & editing, Methodology, Conceptualization. **Bethany A. Kalich:** Writing – review & editing, Writing – original draft, Methodology, Conceptualization. **Seth S. Martin:** Writing – review & editing, Writing – original draft, Methodology, Conceptualization.

## Declaration of competing interest

We wish to draw the attention of the Editor to the following facts which may be considered as potential conflicts of interest and been no significant financial support for this work that could have influenced its outcome. We confirm that the manuscript has been read and approved by all named authors and that there are no other persons who satisfied the criteria for authorship but are not listed. We further confirm that the order of authors listed in the manuscript has been approved by all of us. We understand that the Corresponding Author is the sole contact for the Editorial process (including Editorial Manager and direct communications with the office). He/she is responsible for communicating with the other authors about progress, submissions of revisions and final approval of proofs. We confirm that we have provided a current, correct email address which is accessible by the Corresponding Author. All authors have agreed.
